# Malakoplakia of the ureter: An unusual case

**DOI:** 10.4103/0970-1591.40627

**Published:** 2008

**Authors:** Jayesh V. Dhabalia, Girish G. Nelivigi, Nilesh K. Jain, Manav Suryavanshi, Shal Kakkattil

**Affiliations:** Department of Urology, KEM Hospital and Seth GS Medical College, Parel, Mumbai - 400 012, India

**Keywords:** Malakoplakia, Michaelis-Guttman bodies, von Hansemann cells

## Abstract

Malakoplakia of the ureter is a rare pathological entity. We discuss a 15-year-old girl with malakoplakia of the ureter. She presented with obstructive uropathy associated with left flank pain. Radiological investigations showed left lower ureteric stricture without bladder or kidney involvement. She was treated by excision of terminal ureter and ureteroneocystostomy. Histopathologic examination of the excised specimen showed malakoplakia. Postoperative course was uneventful and on follow-up, she has normal serum creatinine and no recurrence of the disease.

## INTRODUCTION

Malakoplakia is an unusual inflammatory disease affecting all body organs, but chiefly the genitourinary tract. In the genitourinary tract, it mainly affects the bladder. Involvement of the kidneys, ureters, and prostate is less common. In a review of 153 cases of malakoplakia in 1981, Stanton and Maxted found that only 11% had ureteral involvement.[1] After that there have been only been nine reports with majority of them from Japan. So far there has been no case report of isolated ureteral malakoplakia from India. It is possible that many cases are being missed either because the clinicians are not looking out for this entity or the histopathologists are not trained to diagnose malakoplakia. In fact only about 10% of the pathologists could diagnose malakoplakia as seen in the review by Stanton and Maxted. The concern here is that malakoplakia is not a benign urinary tract infection, but a chronic morbid disease with fatal outcome if there is extensive upper tract involvement. This is especially relevant in our country where patient follow-up is poor. We present a 15-year-old girl who presented with malakoplakia and no prior urinary tract infection. This is the youngest such case reported in literature.

## CASE REPORT

A 15-year-old girl presented with left flank pain, fever, and vomiting of 1-month duration. She had history of pyuria 3 years ago and had undergone drainage of left psoas abscess 8 years ago. She had no history of recurrent urinary tract infection. Physical examination showed palpable and tender left kidney with no other remarkable findings.

**Figure 1:**
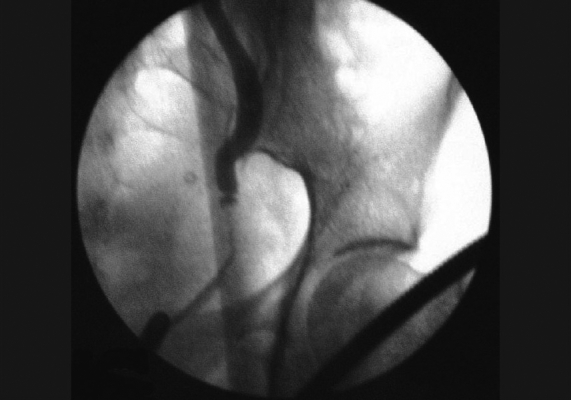
Retrograde Pyelogram showing stricture left lower ureter

Her hemoglobin was 7.5 gm%, serum creatinine was 2.8 mg%, and urine showed 20-25 pus cells and plenty of bacteria per high-power field. Urine culture grew *E. coli*. Ultrasound examination showed left gross hydroureteronephrosis till mid ureter with internal echoes and small right kidney. Despite continuous per urethral catheter drainage and parenteral antibiotics, there was no improvement. Micturating cystourethrogram was normal. On cystoscopy, bladder was normal and retrograde pyelogram showed a stricture measuring 2.5 cm in left lower ureter [Figure 1]. Guide wire could not be negotiated across. In view of the stricture, we suspected genitourinary tuberculosis. However, urine for Polymerase Chain Reaction (PCR) and Acid Fast Bacilli (AFB) for mycobacterium were negative.

**Figure 2:**
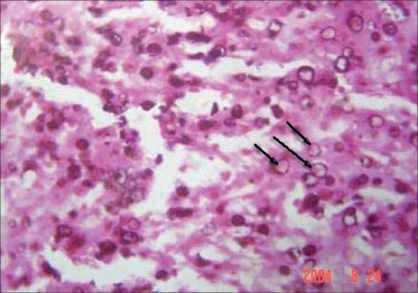
Von Kossa stain showing Michaelis Gutmann bodies

The patient underwent left ureteric reimplantation. The strictured lower ureter was excised and sent for HistoPathology Examination (HPE). The HPE showed Michaelis Gutmann bodies pathognomonic of malakoplakia [Figure 2]. The patient was asymptomatic with normal serum creatinine at 1 year after which she was lost for follow-up.

## DISCUSSION

Malakoplakia is an inflammatory disease that affects the genitourinary tract with a special affinity for bladder. It also affects GIT, bones, and lungs. The patients are usually above the age of 50 years with a significant male predominance. However, in the genitourinary tract, there is a female predominance.[1] The exact pathogenesis is unknown, but it is generally assumed that a combination of chronic bacterial infections in a patient with chronic debility or immunosupression causes this disease. Nearly 90% of the patients have coliform urine infections and 40% have autoimmune disease or some type of immunodeficiency.[1] Witherington *et al*. have hypothesized that diminished monocytic bactericidal activity against *E. coli* is responsible for the unusual immunologic response that causes malakoplakia.[2]

The symptoms of bladder malakoplakia are bladder irritability and hematuria. Isolated upper tract involvement occurs in 15% of the patients. However, involvement of ureter only without kidney involvement occurs very rarely and so far only nine cases have been reported.[3] The exact mechanism of isolated ureteric involvement is unknown, but is thought to be due to a less aggressive variant of the disease.[4] In the involvement of ureter or renal pelvis, the patient may have symptoms manifested due to upper urinary tract obstruction. In cases of renal parenchymal infection, the patient may have fever, flank pain, and a flank mass in association with urinary tract infection. Malakoplakia of the testis may manifest as epididymo-orchitis. Prostatic malakoplakia may manifest as a hard induration on DRE mimicking carcinoma prostate.

Ultrasonography shows hyperechoic renal parenchyma with distortion of central echogenic complex. Excretory Urography shows enlarged kidneys with multiple filling defects with absent hydronephrosis. CT scan shows malakoplakia as hypodense lesions. However, the definite diagnosis is made by biopsy. Microscopically, there are aggregates of large mononuclear phagocytes - the von Hansemann cells admixed with intracellular and extracellular Michaelis-Gutmann bodies all in a scanty connective tissue stroma and infiltrated by varying numbers of lymphocytes and plasma cells. Michaelis-Gutmann bodies are pathognomonic of malakoplakia and are discrete, sharply demarcated intracellular or extracellular ‘calculospherules’ usually with a concentric owl-eye appearance.[5] However, they may not be seen in the early stages of the disease and are not absolutely necessary for the diagnosis.

The initial treatment of malakoplakia consists of prompt treatment of urinary infection and surgery for the affected site. The prognosis of bilateral renal involvement is poor with 100% mortality within 6 months irrespective of the treatment chosen. Unilateral renal or upper tract involvement needs nephrectomy and has good results. It is not known if leaving behind the positive surgical margins following resection has any bearing on the prognosis. Focal involvement of only lower ureter can be treated with local excision and ureteroneocystostomy. Malakoplakia of the lower urinary tract is more benign with a good prognosis. After the initial treatment with antibiotics, if there is a persistence of plaques in the bladder, they can be endoscopically resected and cystoscopically followed up. Use of bethanechol is controversial with conflicting literature on its benefits.
